# Incidence, Treatment, and Survival of Adrenocortical Carcinoma in Denmark 2003-2019

**DOI:** 10.1210/jendso/bvae012

**Published:** 2024-01-30

**Authors:** Jens Pedersen, Anne Elisabeth Jarløv, Åse Krogh Rasmussen, Kirstine Stochholm

**Affiliations:** Department of Medicine, Division of Endocrinology, Herlev and Gentofte Hospital, 2730 Herlev, Denmark; Department of Endocrinology and Metabolism, Rigshospitalet, 2100 Copenhagen, Denmark; Department of Endocrinology and Metabolism, Rigshospitalet, 2100 Copenhagen, Denmark; Department of Internal Medicine and Endocrinology, Aarhus University Hospital, 8200 Aarhus, Denmark

**Keywords:** adrenocortical carcinoma, Denmark, survival, incidence, mortality, retrospective

## Abstract

**Objectives:**

Adrenocortical carcinoma (ACC) is a malignant tumor originating from the adrenal cortex. The aim of the study was to report the incidence of ACC and survival of ACC in Denmark. The secondary objective was to describe the impact of treatment with mitotane on survival.

**Design:**

Retrospective population study of patients diagnosed with ACC between 2003 and 2019 in Denmark.

**Methods:**

Individuals at risk for ACC were identified in the national Danish Health registries, and diagnosis of ACC was confirmed by review of the health records. Data on demographics, presentation, treatment, recurrence, and death was evaluated.

**Results:**

138 patients were included in the study with more females (59.4%) than males (40.6%). Incidence rate was 1.4 per million per year. The incidence rate ratio significantly increased only in females by 1.06 [95% confidence interval (CI): 1.02-1.12] per year. Overall median survival was 1.93 (95% CI: 1.24-3.00) years with no differences between males and females. The proportion of patients treated with mitotane (either as adjuvant treatment or as part of a chemotherapeutic regime) was 72.3%. Survival was significantly decreased in women not treated with mitotane compared to women treated with mitotane (either as adjuvant or as part of a chemotherapeutic regime) hazards ratio .30 (95% CI: .10-.89), adjusted for European Network for the Study of Adrenal Tumours score, age at diagnosis, and year of diagnosis, but survival was unaffected by mitotane treatment in men.

**Conclusion:**

Incidence of ACC in Denmark was 1.4 per million per year and increased in women but not in males during the study period 2003-2019.

Adrenocortical carcinoma (ACC) is a rare malignant tumor originating from the adrenal cortex, with an incidence of ACC of .7 to 2.0 per million per year [1]. More women than men are affected with a ratio of 1.5:1. Most patients are diagnosed in their fourth or fifth decade. Most ACC tumors occur sporadically. However, ACC may be caused by rare hereditary conditions among young patients. The overall prognosis of ACC is poor, with an overall median survival time ranging from 17 months to 104 months [2-5] depending on European Network for the Study of Adrenal Tumours (ENSAT) stage and biological and genetic factors included in the reported cohorts.

Different staging systems have been proposed recently [2, 6]. However, the ENSAT staging is currently recommended by the European Society of Endocrinology [1]. ENSAT 1 is a localized tumor with a diameter of ≤5 cm, ENSAT 2 is a localized tumor with a diameter of >5 cm, ENSAT 3 is a tumor of any diameter with invasion or infiltration into adjacent organs or tissue or lymph node invasion, and ENSAT 4 is a tumor with distant metastasis [7].

The primary treatment of ACC is surgery. Removal of the tumor is recommended for all ENSAT stage 1-3 tumors and can be considered for oligometastatic stage 4 tumors [8]. Stage 4 tumors with no surgical options are treated with systemic chemotherapy, and, according to European guidelines, a regime consisting of etoposide, doxorubicin, cisplatin, and mitotane is recommended [1, 9], although prognosis despite chemotherapy is poor [9].

We present a retrospective study of patient characteristics, treatment, recurrence, and survival of Danish patients diagnosed with ACC during the study period 2003-2019. We hypothesize that the overall incidence rate of ACC has increased during the study period and that more patients are diagnosed with ACC with stage 1 and 2 at the end of the study period compared to the beginning of the study period.

## Methods

### Registries Used for Identification of ACC Cases

Since 1967, all inhabitants in Denmark have had a unique, permanent, and centrally registered social security number. This number is used for identification and allows linkage of data from numerous different registries on an individual level [10].

The Danish National Patient Registry (DNPR) is a nationwide hospital registry, established in 1977 and from 1995 covering all hospital activities, registering all hospital contacts using International Classification of Disease (ICD) version 10 codes [11] and relevant dates. The Danish Pathology Register (DPR) contains data from every specimen sent for pathologist review from 1999 to the present; data are coded according to the Danish version of Systematized Nomenclature of Medicine [12]. The Danish Cancer Registry (DCR), established in 1942, contains data on all patients diagnosed with cancer in Denmark [13]. Reporting to the various registers is mandatory and automated.

### Identification of Individuals at Risk of ACC

To identify all individuals in Denmark at risk of ACC, we retrieved data on all patients registered from 2003 to 2019 with ICD-10 codes C74.0 (malignant neoplasm of the adrenal cortex) and C74.9 (malignant neoplasm of the adrenal gland unspecified) in the DNPR and DCR, as well as all individuals registered in DPR with the codes M83703 (adrenocortical carcinoma), M83707 (adrenocortical carcinoma relapse), or M83709 (adrenocortical carcinoma, primary or metastasis). We chose this strategy of inclusion of some diagnoses with rather low risk of ACC to minimize the number of patients with ACC not identified using the registries.

### Identification of ACC Patients

In Denmark, healthcare is provided by a tax-paid healthcare system, and the Danish Health Authority is the central authority for all hospitals. Only 2 centers (Aarhus University Hospital and Rigshospitalet, Copenhagen University Hospital) are appointed by the Danish Health Authority to treat and follow up on patients with ACC.

Paper and/or electronical health records of all registered individuals with at least 1 ACC code from 1 of the 3 registries were manually reviewed by 2 expert endocrinologists. Only individuals with a verified ACC diagnosis by 1 of the 2 experts were included as patients with ACC in the study. Patients were classified as treated with mitotane or other chemotherapy if treatment was instituted regardless of treatment length. Information extracted from the health records and pathology evaluation of the removed tumor (Table 1) were registered in a REDCap database [14].

**Table 1.** Characteristics and treatment of patients with adrenocortical carcinoma in the period 2003-2019

Demographics		Total sample	Male	Female
Participant characteristics	Number of participants	n = 138	n = 56	n = 82
	Mean age (+/−SD) at enrollment	55.8 years (±16.8)	56.5 years (±15.2)	55.3 years (±17.7)

		Total: patients/patients with available information (%)	Males: patients/patients with available information (%)	Females: patients/patients with available information (%)
Characteristics at diagnosis	Clinical signs of cortisol overproduction*^b^*	41/87 (47.1)	9/32 (28.1)	32/55 (58.2)
	Biochemical signs of cortisol overproduction	38/72 (52.8)	11/28 (39.3)	27/44 (61.4)
	Biochemical signs of androgen overproduction	18/47 (38.3)	4/15 (26.7)	14/32 (43.8)
	Excessive (>.4 mg/day) urine excretion of tetrahydro-11-deoxycortisol	23/27 (85.2)	7/9 (77.8)	16/18 (88.9)
	Signs of metastasis by imaging (yes/no)	51/108 (47.2)	23/43 (53.5)	28/65 (43.1)
Pathology	Tumor size	121/138 (87.7)	47/56 (83.9)	74/82 (90.2)
	- Mean size; cm (SD)	11.8 cm (±5.5)	11.7 cm (±5.6)	11.8 cm (±5.8)
	Weiss score*^a^*	60/106 (56.6)	21/42 (50.0)	39/64 (60.1)
	Weiss ≥6	41/60 (68.3)	14/21 (66.7)	27/39 (69.2)
	Weiss 3-5	19/60 (31.7)	7/21 (33.3)	12/39 (30.8)
	Signs of invasion	91/106 (85.8)	34/42 (81.0)	57/64 (89.1)
	Yes	41/91 (45.1)	14/34 (41.7)	27/57 (47.4
	No	50/91 (54.9)	20/34 (58.8)	30/57 (52.6)
Treatment	Surgical procedure	67/106 (63.2)	24/42 (57.1)	43/64 (67.2)
	Open adrenalectomy	56/67 (83.6)	19/24 (79.2)	37/43 (86.0)
	Laparoscopic adrenalectomy	11/67 (16.4)	5/24 (20.8)	6/37 (16.2)
	Use of adjuvant mitotane	68/94 (72.3)	22/37 (59.5)	46/57 (80.7)
	Treatment with mitotane > 2 years	16/68 (23.5)	6/22 (27.3)	10/46 (21.7)
	Chemotherapy	30/95 (31.6)	10/38 (26.3)	20/57 (35.1)
	EDP/M regime	15/30 (50.0	5/10 (50.0)	10/20 (50.0)
	Sz-M regime	0/30 (.0)	0/10 (.0)	0/10 (.0)
	Other regime	15/30 (50.0)	5/10 (50.0)	10/20 (50.0)
	Macroscopically free resection boundaries	64/75 (85.3)	26/27 (96.3)	38/48 (79.2)
	Adjuvant radio therapy against tumor bed	14/78 (17.9)	4/29 (13.8)	10/49 (20.4)
	Irradiation dose available	10/14 (71.4)	2/4 (50.0)	8/10 (80.0)
	- Irradiation dose	55.5 Gy (±4.2)	54.0 Gy (±0)	55.9 Gy (±4.7)
Course of disease	Patients with known recurrence*^c^*	75/99 (75.8)	31/40 (77.5)	44/59 (74.5)
	Number of deaths as recurrence	24/99 (24.2)	11/40 (27.5)	13/59 (22.0)
	Number of progressive dissease*^c^* (IQR)	1.8 (1;3)	1.8 (1;2)	1.8 (0; 3)
	Mean time to first recurrence*^c^* (IQR)	2.3 years (.3; 3.7)	3.1 years (.2; 5.0)	1.8 years (.6; 2.7)

Abbreviations: EDP/M, etoposide, doxorubicin cisplatin, and mitotane; IQR, interquartile range; Sz-M. streptozotocin and mitotane.

Variables registered from the medical health records.

^*a*^Weiss scores were stratified into scores above 6 or 3-5, as scores above 6 are associated with a more unfavorable prognosis [2].

^*b*^Clinical signs of cortisol overproduction: plethora, moon face, buffalo hump, striae, and ecchymosis.

^*c*^Defined as sign of recurrence, either clinical or using imaging, or death.

Due to the long range of the study period, not all data were available in all patients.

### Exclusion

Individuals without registered health records at either of the 2 national centers for ACCs were excluded, as were individuals where the diagnosis of ACC could not be confirmed by review of the health record by the expert, using following exclusion criteria: (1) removal of adrenal gland without pathology registration of adrenocortical carcinoma; (2) no adrenalectomy and benign radiology characteristics (tumor diameter < 4 cm, tumor diameter > 4 cm and Hounsfield units < 10 or absolute and relative washout >40 and >60, respectively); (3) no tumor detected in the adrenals (no adrenalectomy or no diagnostic imaging of the adrenals) and no biochemical and/or clinical sign of excessive cortisol production; (4) tumor of the adrenal most likely metastasis of another disseminated cancer; (5) other adrenal pathology, for instance neuroblastoma; (6) diagnosis of ACC not during study period; (7) no diagnosis related to adrenals; (8) ACC not likely; (9) missing data.

### Statistics

Continuous data are presented as mean and interquartile ranges; categorical data are presented as numbers and percentages.

Age-adjusted incidence rate was calculated using the European standard population [15], which has previously been used by others [5]. The change in annual incidence ratio of ACC per 1 million from the background population with the same sex was calculated using Poisson regression.

A Cox proportional hazards regression model was used for analysis of recurrence and survival. The analyses were undertaken in all patients who had relevant information. Recurrence was defined as sign of recurrence, either clinical or using imaging, or death, and time at risk started at date of diagnosis and ended at first recurrence, death, or date of last registration in file, whichever came first. Regarding mortality, time at risk started at date of diagnosis and ended at death or date of manual review of health record, whichever came first. Comparisons of ordinal data was calculated using the Mann–Whitney U test. Comparisons of proportions were analysed using Chi-square. The RECORD (The REporting of studies Conducted using Observational Routinely-collected health Data) guideline was followed [16].

### Ethics

The study was registered by the Capital Region of Denmark (P-2020-467), and permission to review health records without patient consent was obtained from the Danish Health Authority (reference: 3-3013-3070/1).

## Results

### Cohort and Incidence

**Figure 1.**
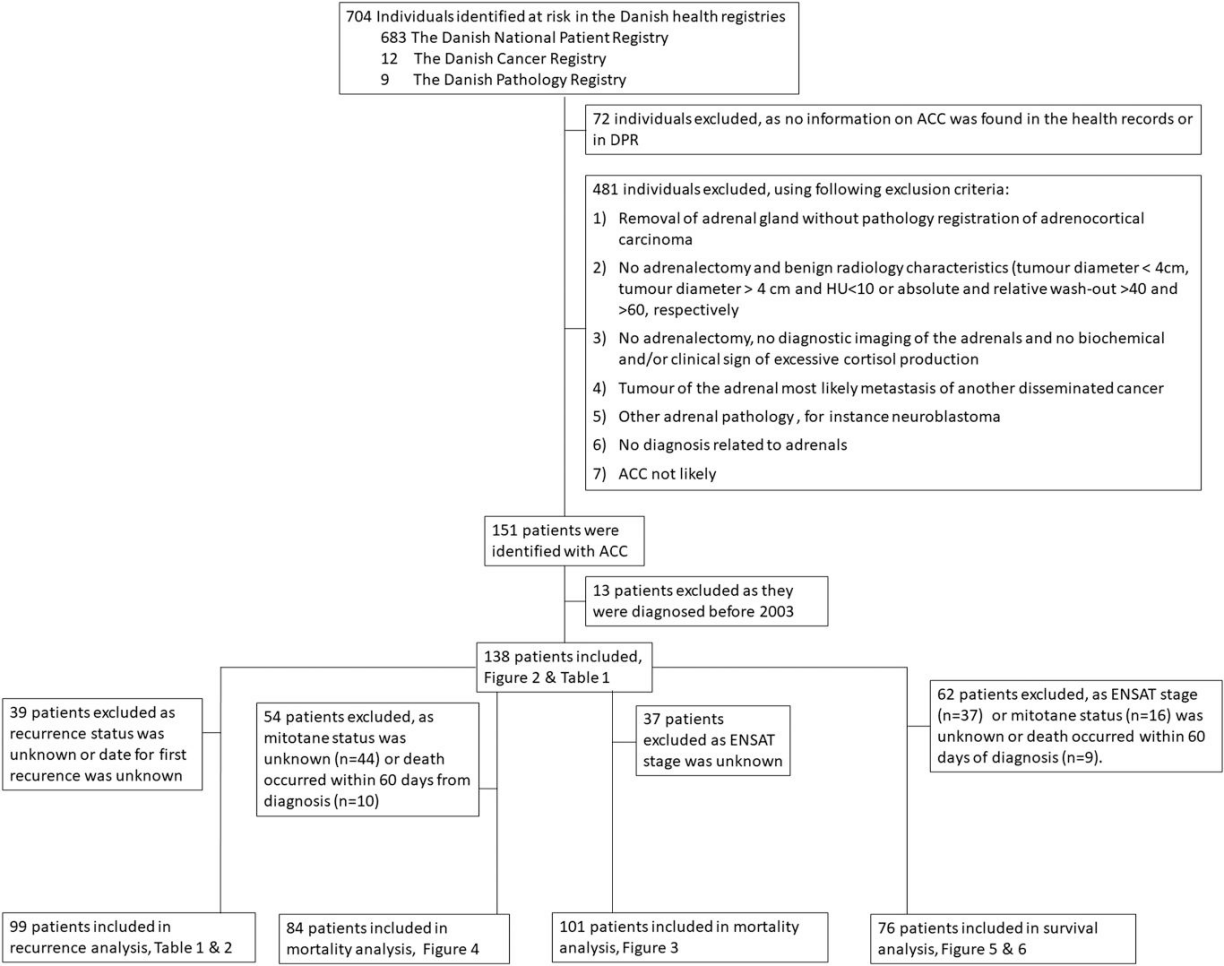
CONSORT diagram of included patients with adrenocortical carcinoma in the different Cox proportional hazard regression analysis.

We identified 704 individuals at risk: 683 individuals registered with the ICD-10 C74.0 or C74.9 in DNPR supplemented with 12 individuals registered with the ICD-10 C74.0 or C74.9 in DCR and a further 9 individuals registered with Systematized Nomenclature of Medicine M83703, M83707, or M83709 in DPR. Seventy-one individuals of the 683 were excluded as there was no pathology record of M83703, M83707, or M83709 in the DPR or any health record available at the 2 national centers. A further 482 individuals were excluded as the diagnosis of ACC could not be confirmed by examination of the health records (42 individuals with adenoma, 47 individuals with pheochromocytoma, 100 individuals with neuroblastoma, 199 individuals with metastasis in the adrenals, 34 individuals with other adrenal pathology, and 60 individuals with no diagnosis related to adrenals) (Fig. 1).

We verified the ACC diagnosis in 151 patients, whereof 138 were diagnosed with ACC during the study period from 2003 to 2019. During the study period, the age-adjusted incidence rate was 1.4/million/year. The incidence rate ratio increased significantly in women [1.06 (95% confidence interval [CI]: 1.02-1.12)] but not in men [.98 (95% CI: .93-1.04)].

### Presentation, Tumor Size, Stage at Diagnosis and Treatment

The mean age at time of the diagnosis with ACC was 55.8 (±16.8) years, and more females (59.4%) than males (40.6%) were diagnosed with ACC, corresponding to a ratio of 1.5.

The number of patients with clinical signs of cortisol/androgen excess was higher in females compared to men (*P* < .001, Table 1). Furthermore, 23 of the 27 patients (85.2%) who had urine steroid metabolites analyzed at the time of diagnosis had increased urine excretion of 11-deoxycortisol metabolite tetrahydro-11-deoxycortisol. All available clinical and biochemical characteristics are presented in Table 1.

ENSAT stages were available in 101 patients, and the distribution of different ENSAT stages in our cohort is shown in Table 2. Most patients had ENSAT stage 4 (n = 51/101, 50.5%) at diagnosis, and only 4/101 patients (3.9%) were diagnosed with an ENSAT stage 1 tumor (Table 2). The proportion of different ENSAT stages at time of diagnosis did not change from 2003 to 2019 (*P* = .96).

**Table 2.** ENSAT stage at time of diagnosis and time at risk defined as time from date of diagnosis and ended at first recurrence, death, or at date of last registration in file, whichever came first

Distribution of number of all patients	ENSAT 1, n = 4	ENSAT 2, n = 30	ENSAT 3, n = 16	ENSAT 4, n = 51	ENSAT unknown, n = 37
Number of patients with known recurrence status (%)	4 (3.9)	28 (29.7)	14 (15.8)	38 (50.5)	15 (26.8)
Sex distribution male/female	2/2	11/17	4/10	18/20	5/10
Mean time at risk (IQR)	5.2 years (3.6; 6.9)	4.2 years (1.6; 6.5)	3.1 years (.4; 4.6)	.7 years (.1; .8)	2.6 years (.1; 5.0)

Abbreviations: ENSAT, European Network for the Study of Adrenal Tumours; IQR, interquartile range.

Most patients underwent unilateral adrenalectomy (n = 106/136, 77.9%), and none of the patients had bilateral adrenalectomy. Only a subset of patients had a laparoscopic procedure (n = 11/67, 16.4%). All patients with stage 1 to 3 were adrenalectomized, but only 25 out of 49 (51.0%) of patients with stage 4 tumors were adrenalectomized. Information about resection boundaries were collected from the surgery description recorded in the health records. Forty-four out of 46 patients (95.6%) with ENSAT stage 1 to 3 had macroscopically free resection boundaries free of tumor, whereas only 10 out of 19 of patients (52.6%) with ENSAT stage 4 had macroscopically free resection boundaries.

Treatment with mitotane was used overall in 68 out of 94 patients (72.3%). Two out of 4 patients (50%) with ENSAT 1 were treated with mitotane and 33 out of 44 patients (75.0%) with stage 2 or 3 disease received treatment with mitotane. Of these 33 patients, 19 patients were treated with mitotane within 60 days following adrenalectomy, indicating an adjuvant treatment. The proportion of patients with ENSAT stage 1 to 3 treated with mitotane did not change during the study period (*P* = .83). Twenty-six out of 37 patients (70.3%) with ENSAT stage 4 disease (advanced disease) were treated with mitotane.

We found no difference in mean ENSAT stage in patients who were treated with mitotane [3.0 (95% CI: 2.7-3.2), n = 59] and patients who were not treated [2.6 (95% CI: 2.1-3.1), n = 17], Mann–Whitney, *P* = .18. Further, among patients treated with mitotane, the mean ENSAT stage was similar in men: 2.9 (95% CI: 2.4-3.4), and in women: 3.0 (95% CI: 2.7-3.3) (Mann–Whitney, *P* = .71). Among patients not treated with mitotane, the mean ENSAT stage was similar in men, 2.6 (95% CI: 1.8-3.3) and in women, 2.6 (95% CI: 1.8-3.4) (Mann–Whitney, *P* = .99).

Thirty patients were treated with systemic chemotherapy; 15 patients were treated with a etoposide, doxorubicin, cisplatin, and mitotane regime. No patients were treated with the streptozotocin and mitotane regime. Two patients were treated with cisplatinum, etoposide, and mitotane; 3 patients were treated with a regime combining cisplatinum and docetaxel. However, 10 patients were treated with an unknown protocol not registered in the electronic health records. However, 8 of the patients treated with an unknown protocol were also treated with mitotane.

### Progression

Thirty-six patients had unknown recurrence status, and 3 patients had unknown date for first recurrence and were not included in the recurrence analysis. Mean time from diagnosis to first progression or death or last visit, whichever came first, for 99 patients with known recurrence status and known date for first recurrence was 2.5 (interquartile range: .2; 4.2) years. Mean time at risk for recurrence, as defined earlier, divided into ENSAT stages is shown in Table 2.

Twenty-four out of 99 patients had no progression and were alive at the time of last registration in the health record. None of these patients were diagnosed with an ENSAT 4 stage tumor; however, information about stage at diagnosis was missing in 4 of these cases. Twenty-two patients (91.7%) who survived with no progression had macroscopically free resection boundaries, and 2 patients who survived with no progression had unknown macroscopically resection boundaries. Among the 75 patients with 1 or more progressions or death, 37 (49.3%) patients had macroscopically free resections boundaries, whereas 9 (12.0%) patients did not have macroscopically free resection boundaries and 29 (38.7%) patients had unknown macroscopically resection boundaries, significantly different from the distribution in the patients without recurrence (χ^2^ test, *P* < .002).

### Survival

**Figure 2.**
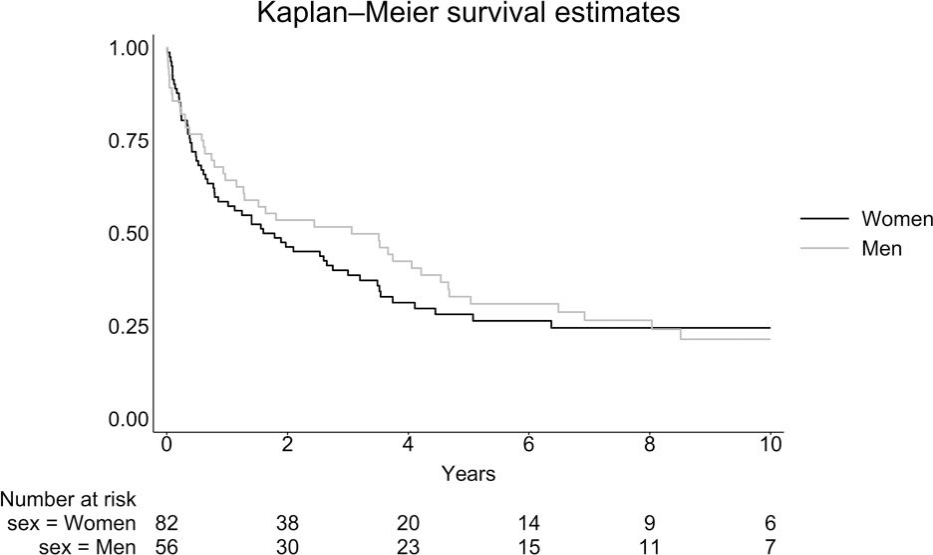
Survival in 138 patients with adrenocortical carcinoma diagnosed between 2003 and 2019 in Denmark divided into women and men using a Kaplan–Meier plot.

Survival data were available for all 138 patients included, and 102 patients out of 138 patients were deceased at the time of extraction of information from the health records. The 2-year survival rate was 46.3% (38/82) for women and 53.6% (30/56) for men. Median overall survival for both men and women (n = 138) was 1.93 (95% CI: 1.24; 3.00) years. Median overall survival for women (n = 82) was 1.69 (95% CI: .79; 2.75) years and for men (n = 56) 2.75 (95% CI: 1.17; 4.36) years. The mortality in all 138 patients was similar in men and women with a hazard ratio (HR) of .87 (.59; 1.30) (Fig. 2).

**Figure 3.**
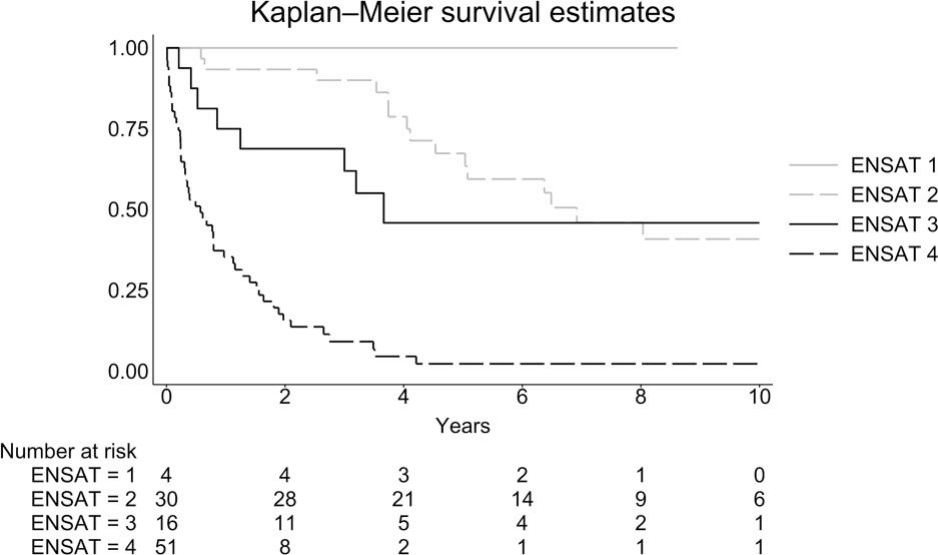
Survival in 101 patients with adrenocortical carcinoma with available information on ENSAT score using a Kaplan–Meier plot.

Information on ENSAT [1-4] was available in 101 (73.2%) patients, and survival increased 3.3 years (95% CI: 2.4-4.6) with every decrease in ENSAT score (Fig. 3).

**Figure 4.**
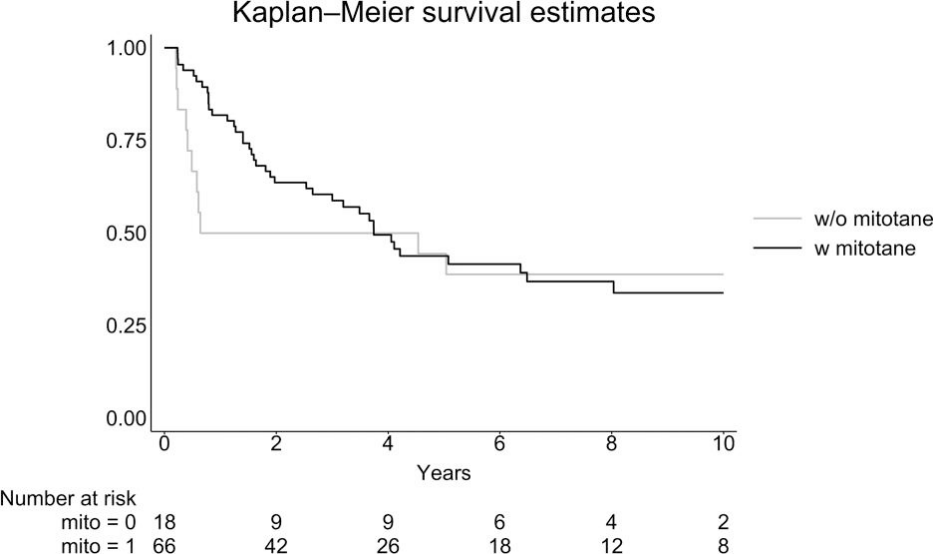
Survival in 84 patients diagnosed with adrenocortical carcinoma with available information on mitotane treatment, divided into without (w/o) mitotane or with (w) mitotane using a Kaplan–Meier plot. All patients were alive more than 60 days from diagnosis.

Among the 138 patients, information on treatment with mitotane (yes/no) was available in 94 (68.1%). The 2 patients with no information about mitotane treatment (adjuvant or part of chemotherapeutic regime) were not included in the analysis of mitotane effects (n = 44). Using sex as a covariate, survival was significantly increased in patients treated with mitotane compared to those not treated with mitotane with a HR of .50 (95% CI: .28-.87), *P* < .05. Including only patients who survived more than 60 days from diagnosis (n = 84), the HR was .76 (95% CI:.38-1.51), *P* = .43 for patients treated with mitotane compared to untreated patients (Fig. 4).

Information on treatment with mitotane (yes/no) and ENSAT stage 1 to 4 was available in 85 (61.6%) patients. Nine of these patients died within 60 days from diagnosis; they were all diagnosed with an ENSAT 4 tumor; 7 were not treated with mitotane and 2 were treated with mitotane. The patients who died within 60 days from diagnosis were excluded from all following calculations. The ENSAT stage was significantly higher in those who died more than 60 days from diagnosis [mean ENSAT stage 3.3 (95% CI: 3.0-3.5), n = 47] compared to those who survived [mean ENSAT stage 2.3 (95% CI: 2.0-2.6), n = 29, Mann–Whitney, *P* < .001].

**Figure 5.**
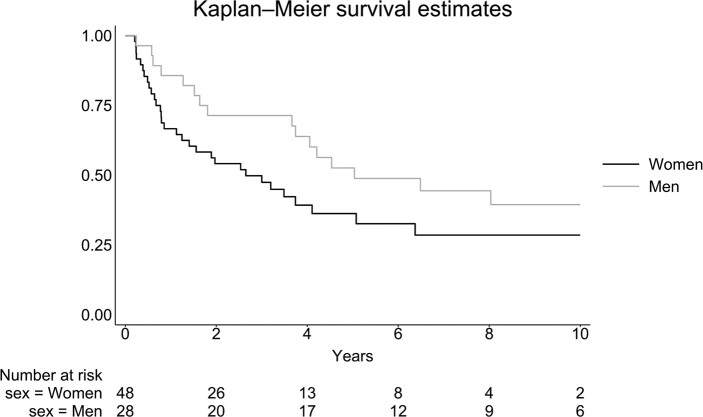
Survival in 76 patients with adrenocortical carcinoma with available information on ENSAT score and mitotane treatment, divided into women and men using a Kaplan–Meier plot. All patients were alive more than 60 days from diagnosis. Abbreviations: ENSAT, European Network for the Study of Adrenal Tumours.

Using ENSAT stage and treatment with mitotane (either as adjuvant or as part of a chemotherapeutic regime) as covariates, survival in women with ACC was significantly decreased compared to men with ACC, with a HR of .42 (95% CI: .21-.81) (Fig. 5). Using ENSAT stage and adjuvant treatment with mitotane as covariates, no difference in survival between women and men was found [HR for death was 1.0 (.6-1.6)].

**Figure 6.**
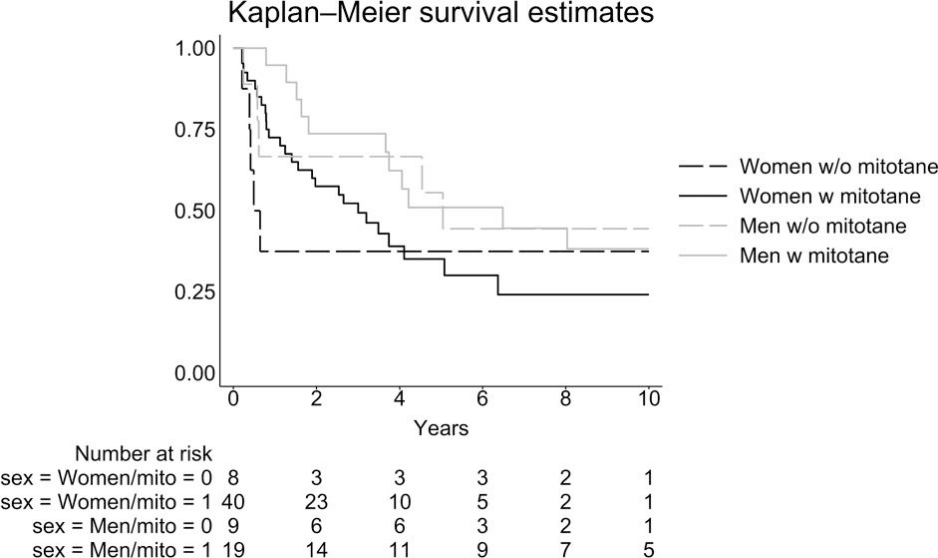
Survival in 76 patients with adrenocortical carcinoma with available information on ENSAT score and mitotane treatment, divided into without (w/o) mitotane or with (w) mItotane and into women and men using a Kaplan–Meier plot. All patients were alive more than 60 days from diagnosis. Abbreviations: ENSAT, European Network for the Study of Adrenal Tumours.

Comparing those not treated with mitotane to those treated, survival was significantly reduced in women, HR .30 (95% CI: .10-.89), but unaffected in men, .69 (95% CI: .23-2.13) (Fig. 6).

## Discussion

We found a crude incidence rate of ACC in Denmark in line with the rates previously reported from other countries and consorts [5, 17]. Furthermore, our findings demonstrated a slightly increase in the yearly incidence among women during the study period. The proportion of lower stage tumors did not change over the study period, which could be expected with the increased use of diagnostic imaging scans during the period. However, the rapid development of ACC might be an explanatory factor. The median overall survival of 1.9 years might seem low, but our cohort included a large proportion of patients with ENSAT stage 3 to 4, which will have a negative impact on the overall survival time. Overall, the clinical characteristics of our cohort and the overall prognosis are like previous reports from other countries and consorts [2, 17].

We saw a reduced survival in women compared to men in patients surviving more than 60 days from diagnosis and with known ENSAT stage, which is in contrast to a previous study by Else et al [18] who found a HR for death of 1.4 for men in a univariate analysis, but where sex was not a prognostic factor in a multivariate analysis combining sex with size of tumor, cortisol secretion, androgen secretion, and stage. Apart from the larger proportion of patients with ENSAT 4 tumors in our cohort, the 2 cohorts are quite similar regarding sex and age distribution as well as cortisol excess [18].

The use of mitotane for low stage tumors with a low risk of recurrence has been debated for a long time [19]. The ADIUVO trial was specifically designed to address this question, by randomizing patients with ENSAT 1 to 3, R0, and Ki67 < 10% to either adjuvant mitotane or active surveillance. The study did not show any effect of mitotane treatment in patients with a low risk of recurrence [20]. Recently, Elhassan et al reported from a large retrospective multicenter study that only patients with ENSAT 3 and Ki67 > 20% would benefit from adjuvant mitotane treatment, and the authors propose adjuvant mitotane to be offered for patients with the new proposed staging of S-GRAS 4-5 [2]. In our study we found a significant reduced HR for death in women treated with mitotane (either as adjuvant treatment or as part of a chemotherapeutic regime) compared to the women who were not treated with mitotane. We did not find this result in men. To our knowledge, this is the first time that a sex-specific result is reported by treatment with mitotane. In fact, no sex-specific outcome was observed in a recent retrospective study by Allegra et al [21]. We speculate that the women included in our cohort likely had more aggressive disease than the men, and women may have had more benefit from treatment with mitotane.

The age-adjusted incidence of 1.4/million/year is comparable to previous studies reporting an incidence between .7 and 2/million/year. However, the Danish age-adjusted incidence rate is higher than reported in a recent study from Finland [17] reporting an incidence of .9/million/year and a recent study from the Netherlands [5] reporting an incidence of 1.0/million/year. Both countries are comparable to Denmark, and both countries also have national cancer registries. However, both studies only used data from their respective cancer registries to calculate the incidence rates. In our study we used data from multiple registries and verified the diagnosis in the health records from the only 2 centers in Denmark that are involved in the treatment of ACC. Thus, we achieved a more complete data collection and likely identified more cases. Because ACC is very rare, even a small increase in identified cases will have a relatively high impact on the calculated incidence rate. The true incidence in Denmark might be even higher than presented, as we excluded 72 individuals without any information about adrenalectomy or follow-up at the 2 ACC centers. A few patients diagnosed elsewhere in Denmark with end-stage ACC and limited survival might be palliated locally and never transferred to 1 of the 2 centers, thereby falsely reducing the estimated incidence and improving survival for the remaining.

A recent report from the RARECARE project [22] described an incidence of 2/million/year, but this incidence also included pheochromocytomas and therefore is not comparable to our incidence of ACC.

In contrast to the study by Kerkhofs et al [5] who found a stable incidence rate, we found a small although significant increase in the incidence rate in women. The slight increase in incidence rate in women could be due to increased use of diagnostic imaging of the abdomen [23]; however, this does not explain the difference in men and women.

The reported proportion of patients with signs of Cushing syndrome (47.1%) was in line with findings of other studies reporting a proportion of Cushing syndrome at diagnosis of ACC ranging from 32% to 63% [2, 6, 17, 24, 25]. Recently, the EURINE-ACT study included tetrahydro-11-deoxycortisol (THS) in the malignant steroid fingerprint [26]. Interestingly, we observed that a very high proportion of patients examined for excretion of steroid metabolites had increased excretion of THS. We believe that the high proportion of patients with high urine excretion of THS indicates that most ACC tumors do produce some steroids, regardless of the smaller proportion of patients presenting with clinical signs of Cushing syndrome.

In agreement with other studies [5, 17, 24, 25] and the most recent guideline [27], the majority of patients (77.9%) underwent adrenalectomy, underlining that surgery is the primary treatment for ACC.

About 85% of the patients had macroscopically free resection boundaries in accordance with the findings of others [2, 25]. However, we were unable to extract information about microscopically resection boundaries, and this is of course a limitation of our study, as it has been shown that microscopic free resection boundary is a major prognostic factor for recurrence-free survival [2]. We found that patients with no progression all had macroscopically free resection boundaries and that some patients with progression had macroscopically free resection boundaries, indicating that macroscopically free resection boundaries are necessary but not sufficient for recurrence-free survival.

Our study has limitations. The study is not randomized regarding mitotane, and the retrospective design is a major limitation as data available is not uniform for each case, and the data available from patients diagnosed in the early part of the study period are fewer than data from patients diagnosed in the later part of the study period, thus introducing a possible time bias. The missing information about microscopically resection boundaries may also be a limitation of our study. Missing patients is another drawback.

The strength of our study is the unique Danish personal identification number that enables identification of individuals linking the Danish national health registries and the electronical health records. Another strength of our study is that data extraction was done by a combination of extracts from health registries and health records.

In conclusion, we have found that the incidence of ACC in Denmark is 1.4/million/year, and the incidence was slightly increasing among women in the study period. No change in tumor staging in the study period was seen. The proportion of ENSAT stage 4 tumors were higher than expected, and we speculate that more awareness of Cushing syndrome could help detect tumors at an earlier stage. The overall survival of patients with ACC depended on biological sex and the stage of the tumor at diagnosis. Furthermore, we have in our observational data found indications of a beneficial effect of mitotane on survival only in women. We speculate that this reflects that the women included in our study had more advanced disease. However, due to the small numbers and the fact that this observation is based on purely retrospective data, this should of course be interpreted with caution, and this finding should be confirmed in future studies.

## Data Availability

Restrictions apply to the availability of all data generated and analyzed during this study to preserve patient confidentiality. The corresponding author will on request detail the restrictions and any conditions under which access to some data may be provided.

## References

[1] Fassnacht M, Dekkers OM, Else T, et al European society of endocrinology clinical practice guidelines on the management of adrenocortical carcinoma in adults, in collaboration with the European network for the study of adrenal tumors. Eur J Endocrinol. 2018;179(4):G1‐G46. 30299884 10.1530/EJE-18-0608

[2] Elhassan YS, Altieri B, Berhane S, et al S-GRAS score for prognostic classification of adrenocortical carcinoma: an international, multicenter ENSAT study. Eur J Endocrinol. 2022;186(1):25‐36. 10.1530/EJE-21-0510 PMC8679848 34709200

[3] Tran TB, Postlewait LM, Maithel SK, et al Actual 10-year survivors following resection of adrenocortical carcinoma: adrenocortical carcinoma survivors. J Surg Oncol. 2016;114(8):971‐976. 27633419 10.1002/jso.24439 PMC5278771

[4] Arnon J, Grozinsky-Glasberg S, Oleinikov K, et al Prognostic factors in advanced adrenocortical carcinoma: summary of a national referral center's 20 years of experience. J Endocr Soc. 2022;6(9):bvac112. 35949453 10.1210/jendso/bvac112 PMC9354968

[5] Kerkhofs TMA, Verhoeven RHA, Van der Zwan JM, et al Adrenocortical carcinoma: a population-based study on incidence and survival in The Netherlands since 1993. Eur J Cancer. 2013;49(11):2579‐2586. 23561851 10.1016/j.ejca.2013.02.034

[6] Libé R, Borget I, Ronchi CL, et al Prognostic factors in stage III–IV adrenocortical carcinomas (ACC): an European Network for the Study of Adrenal Tumor (ENSAT) study. Ann Oncol. 2015;26(10):2119‐2125. 26392430 10.1093/annonc/mdv329

[7] Lughezzani G, Sun M, Perrotte P, et al The European network for the study of adrenal tumors staging system is prognostically superior to the international union against cancer-staging system: a North American validation. Eur J Cancer. 2010;46(4):713‐719. 20044246 10.1016/j.ejca.2009.12.007

[8] Srougi V, Bancos I, Daher M, et al Cytoreductive surgery of the primary tumor in metastatic adrenocortical carcinoma: impact on patients’ survival. J Clin Endocrinol Metab. 2022;107(4):964‐971. 34850915 10.1210/clinem/dgab865 PMC9122637

[9] Fassnacht M, Terzolo M, Allolio B, et al Combination chemotherapy in advanced adrenocortical carcinoma. N Engl J Med. 2012;366(23):2189‐2197. 22551107 10.1056/NEJMoa1200966

[10] Schmidt M, Pedersen L, Sørensen HT. The Danish civil registration system as a tool in epidemiology. Eur J Epidemiol. 2014;29(8):541‐549. 24965263 10.1007/s10654-014-9930-3

[11] Schmidt M, Schmidt SAJ, Sandegaard JL, Ehrenstein V, Pedersen L, Sørensen HT. The Danish national patient registry: a review of content, data quality, and research potential. Clin Epidemiol. 2015;7: 449‐490. 26604824 10.2147/CLEP.S91125 PMC4655913

[12] Bjerregaard B, Larsen OB. The Danish pathology register. Scand J Public Health. 2011;39(7_suppl):72‐74. 21775357 10.1177/1403494810393563

[13] Gjerstorff ML . The Danish cancer registry. Scand J Public Health. 2011;39(7_suppl):42‐45. 21775350 10.1177/1403494810393562

[14] Harris PA, Taylor R, Minor BL, et al The REDCap consortium: building an international community of software platform partners. J Biomed Inform. 2019;95:103208. 31078660 10.1016/j.jbi.2019.103208 PMC7254481

[15] Waterhouse J, Muir C, Correa P, Powel J. Cancer incidence in five continents. Lyon: IARC. 1976;3:456.

[16] Benchimol EI, Smeeth L, Guttmann A, et al The REporting of studies conducted using observational routinely-collected health data (RECORD) statement. PLoS Med. 2015;12(10):e1001885. 26440803 10.1371/journal.pmed.1001885 PMC4595218

[17] Kostiainen I, Hakaste L, Kejo P, et al Adrenocortical carcinoma: presentation and outcome of a contemporary patient series. Endocrine. 2019;65(1):166‐174. 30980285 10.1007/s12020-019-01918-9 PMC6606857

[18] Else T, Williams AR, Sabolch A, Jolly S, Miller BS, Hammer GD. Adjuvant therapies and patient and tumor characteristics associated with survival of adult patients with adrenocortical carcinoma. J Clin Endocrinol Metab. 2014;99(2):455‐461. 24302750 10.1210/jc.2013-2856 PMC3913818

[19] Fassnacht M, Libé R, Kroiss M, Allolio B. Adrenocortical carcinoma: a clinician's update. Nat Rev Endocrinol. 2011;7(6):323‐335. 21386792 10.1038/nrendo.2010.235

[20] Terzolo M, Fassnacht M, Perotti P, et al Adjuvant mitotane versus surveillance in low-grade, localised adrenocortical carcinoma (ADIUVO): an international, multicentre, open-label, randomised, phase 3 trial and observational study. Lancet Diabetes Endocrinol. 2023;11(10):720‐730. 37619579 10.1016/S2213-8587(23)00193-6 PMC10522778

[21] Allegra S, Puglisi S, Brescia I, et al Sex differences on mitotane concentration and treatment outcome in patients with adrenocortical carcinoma. Life. 2021;11(3):266. 33807024 10.3390/life11030266 PMC8004922

[22] van der Zwan JM, Mallone S, van Dijk B, et al Carcinoma of endocrine organs: results of the RARECARE project. Eur J Cancer. 2012;48(13):1923‐1931. 22361014 10.1016/j.ejca.2012.01.029

[23] Smith-Bindman R, Miglioretti DL, Larson EB. Rising use of diagnostic medical imaging in A large integrated health system. Health Aff. 2008;27(6):1491‐1502. 10.1377/hlthaff.27.6.1491 PMC2765780 18997204

[24] Lim JS, Lee SE, Kim JH, Kim JH. Characteristics of adrenocortical carcinoma in South Korea: a registry-based nationwide survey. Endocr Connect. 2020;9(6):519‐529. 32438344 10.1530/EC-20-0196 PMC7354716

[25] Abiven G, Coste J, Groussin L, et al Clinical and biological features in the prognosis of adrenocortical cancer: poor outcome of cortisol-secreting tumors in a series of 202 consecutive patients. J Clin Endocrinol Metab. 2006;91(7):2650‐2655. 16670169 10.1210/jc.2005-2730

[26] Bancos I, Taylor AE, Chortis V, et al Urine steroid metabolomics for the differential diagnosis of adrenal incidentalomas in the EURINE-ACT study: a prospective test validation study. Lancet Diabetes Endocrinol. 2020;8(9):773‐781. 32711725 10.1016/S2213-8587(20)30218-7 PMC7447976

[27] Fassnacht M, Assie G, Baudin E, et al Adrenocortical carcinomas and malignant phaeochromocytomas: ESMO–EURACAN clinical practice guidelines for diagnosis, treatment and follow-up. Ann Oncol. 2020;31(11):1476‐1490. 32861807 10.1016/j.annonc.2020.08.2099

